# PTPRE promotes gastric cancer cell resistance to 5-fluorouracil by inhibiting ferroptosis via the Src/FAK/TRIB3 axis

**DOI:** 10.1371/journal.pone.0351846

**Published:** 2026-06-18

**Authors:** Ming Liu, Xiaomei Liao, Fangchao Wang, Pengqing Jiao, Zhongkai Wang

**Affiliations:** 1 Department of Radiation Oncology, The Fourth Hospital of Hebei Medical University, Shijiazhuang, China; 2 Department of Pain and Rehabilitation, The Fourth Hospital of Hebei Medical University, Shijiazhuang, China; 3 Department of Rheumatology and Immunology, The Fourth Hospital of Hebei Medical University, Shijiazhuang, China; University of Diyala College of Medicine, IRAQ

## Abstract

Acquired drug resistance is a major cause of failure in gastric cancer treatment. The protein tyrosine phosphatase receptor type E (PTPRE) plays an oncogenic role in certain tumours; however, its function in chemotherapy-resistant gastric cancer remains unclear. Therefore, we analysed PTPRE expression in gastric cancer using The Cancer Genome Atlas. In vitro experiments were then conducted to investigate the effects of PTPRE on the resistance of cancer cells to 5-fluorouracil (5-FU) and its potential mechanisms. The results were further validated in vitro using xenograft studies. We found that PTPRE is upregulated in gastric cancer tissues and participates in the induction of 5-FU resistance. Mechanistic studies revealed that PTPRE suppressed ferroptosis in gastric cancer cells and promoted 5-FU resistance by activating the Src/FAK pathway to upregulate TRIB3 expression. In summary, PTPRE suppresses ferroptosis in gastric cancer cells via the Src/FAK/TRIB3 signalling pathway, thereby inducing 5-FU resistance. Intervention with PTPRE and its downstream targets may represent a potential approach for the clinical treatment of 5-FU-resistant gastric cancer.

## Introduction

Gastric cancer, a common digestive system tumour, consistently ranks among the top five cancers globally in terms of both incidence and mortality rates [1]. Although gastric cancer prevention and control have recently improved, the global situation remains critical. The lifetime incidence of gastric cancer in the 2008–2017 birth cohort is projected to reach 15.6 million cases [2]. Asia currently has the largest gastric cancer burden, accounting for approximately two-thirds of all cases worldwide [2]. Specifically, among the cohort born between 2008 and 2017, China and India alone are expected to account for 6.5 million lifetime gastric cancer cases in the absence of changes to current gastric cancer control measures [2]. Although new therapeutic approaches and standardised treatment protocols have extended the survival of patients with gastric cancer, most patients ultimately succumb to treatment failure. Advanced therapeutic methods are urgently needed for clinical application to improve patient outcomes.

Acquired drug resistance is a major cause of failure in gastric cancer treatment. Multiple factors, such as gene mutations [3], enhanced drug efflux [4], and DNA damage repair [5], can contribute to this phenomenon. Ferroptosis is a form of cell death induced by iron-dependent lipid peroxidation [6,7]. Unlike apoptosis, necrosis, autophagy, and pyroptosis, ferroptosis is considered a novel form of programmed cell death [6,7]. According to recent studies, ferroptosis induction could overcome resistance to cancer immunotherapy [8,9] and chemotherapy [10,11]. Therefore, ferroptosis induction through relevant targets may represent a potential strategy for overcoming resistance to gastric cancer treatment.

Protein tyrosine phosphatase receptor type E (PTPRE) is a member of the protein tyrosine phosphatase family that primarily regulates the phosphorylation levels of substrate proteins [12,13]. Increasing evidence suggests that abnormal PTPRE activation is closely linked to multiple tumour progression pathways, including tumour cell proliferation [14], metastasis [15,16], and epithelial-mesenchymal transition [14]. Recent studies revealed that PTPRE mediates chemotherapy resistance in retinoblastoma via the AKT and ERK1/2 pathways [17], and it has been implicated in sorafenib resistance in hepatocellular carcinoma [18]. However, it remains unclear whether PTPRE contributes to chemotherapy resistance in gastric cancer cells by regulating ferroptosis, which warrants further investigation.

## Materials and Methods

### Cell culture

Human gastric cancer cell lines MKN-45, HGC-27, and NCI-N87 were obtained from the Cell Bank of the Chinese Academy of Sciences (Shanghai, China). MKN-28 and HS 746T cells were purchased from Bena Culture Collection (Beijing, China). NCI-N87 and MKN-28 cells were cultured in RPMI 1640 medium (Thermo Fisher Scientific, Waltham, MA, USA) supplemented with 10% fetal bovine serum (FBS; GIBCO BRL, Grand Island, NY, USA). MKN-45 cells were maintained in RPMI 1640 medium containing 20% FBS. HGC-27 and HS 746T cells were grown in DMEM (Thermo Fisher Scientific) supplemented with 10% FBS. All cells were incubated at 37 °C in a humidified atmosphere containing 5% CO₂. Cells were passaged at a 1:2 ratio when they reached 70–80% confluence.

### Bioinformatics analysis

The Birmingham Cancer Data Analysis Portal (https://ualcan.path.uab.edu/) at the University of Alabama was used to conduct a comparative analysis of *PTPRE* expression in normal gastric (n = 34) and gastric cancer (n = 415) tissues obtained from The Cancer Genome Atlas (TCGA) database.

### Transfection

Small interfering RNA (siRNA) targeting PTPRE, TRIB3, and non-targeting control siRNA were purchased from Genomeditech (Shanghai, China). Transfection was performed with Lipofectamine 2000 (Thermo Fisher Scientific, Waltham, MA, USA) at 50 nM siRNA final concentration. Briefly, cells were seeded in 6-well plates to 50%–60% confluence; siRNA and Lipofectamine 2000 were separately diluted in Opti-MEM (Thermo Fisher Scientific), incubated for 5 min each, then mixed and incubated for another 20 min before being added to cells. Medium was replaced after 8 h, and cells were harvested 48 h post-transfection.

PTPRE overexpression plasmid (pcDNA3.1-PTPRE) and empty vector (pcDNA3.1) were from GenePharma (Shanghai, China). Plasmid transfection (2 μg per well) was conducted as above with Lipofectamine 2000. Cells were collected 48 h post-transfection for detection. Stable PTPRE-expressing cell lines were screened with 2 μg/mL puromycin (Genomeditech, Shanghai, China) 48 h after plasmid transfection. Selection medium was refreshed every 2–3 days for 2 weeks; stable clones were picked, expanded, and verified by Western blot.

### Western blotting

Total protein was separated by 10% sodium dodecyl sulphate-polyacrylamide gel electrophoresis and transferred onto polyvinylidene fluoride (PVDF) membranes (Solarbio, Beijing, China). After blocking with 1% bovine serum albumin (BSA; Aladdin Scientific, Shanghai, China) in Tris-buffered saline with Tween-20 (TBST) at room temperature for 1 h, the PVDF membrane was first incubated with primary antibodies at 4 °C overnight. The primary antibodies used were as follows: Rabbit anti-GPX4 (Cat. No. 52455S, 1:1000 dilution), rabbit anti-SLC7A11 (Cat. No. 98051S, 1:1000 dilution), rabbit anti-TRIB3 (Cat. No. 43043S, 1:1000 dilution), rabbit anti-p-FAK (Cat. No. 8556S, 1:1000 dilution), and rabbit anti-p-Src (Cat. No. 6943S, 1:1000 dilution; Cell Signalling Technology, Danvers, MA, USA); Mouse anti-TRIB3 (Cat. No. 43043S, 1:300 dilution), mouse anti-PTPRE (Cat. No. sc-515692, 1:500 dilution; Santa Cruz Biotechnology, Santa Cruz, CA, USA); Rabbit anti-GAPDH (Cat. No. 10494–1-AP, 1:1000 dilution), rabbit anti-FAK (Cat. No. 12636–1-AP, 1:1000 dilution), and rabbit anti-Src (Cat. No. 11097–1-AP, 1:1000 dilution; Proteintech, Wuhan, China). After primary antibody incubation, the membrane was washed three times with TBST (5 min per wash) at room temperature. Subsequently, the membrane was incubated with horseradish peroxidase-conjugated goat anti-rabbit IgG (H + L; Cat. No. RGAR001, 1:2000 dilution; Proteintech, Wuhan, China) at room temperature for 1 h. Following secondary antibody incubation, the membrane was washed again three times with TBST (5 min per wash) to remove unbound secondary antibodies before chemiluminescent detection. Protein bands were visualised using an enhanced chemiluminescence reagent (Bio-Rad, Hercules, CA, USA) and quantified using ImageJ software.

### Cell counting kit (CCK)-8 assay

Logarithmic phase cell suspensions (5 × 10⁴ cells/mL) were inoculated at 100 μL/well into a 96-well plate. Cells were treated with 2–512 μM 5-fluorouracil (5-FU) for 72 h, with pretreatment using Ferrostatin-1 (Fer-1), a specific inhibitor of ferroptosis (MedChem Express, Monmouth Junction, NJ, USA), when necessary. Cell viability was determined using a CCK-8 assay kit (Solarbio) according to the manufacturer’s instructions.

### Colony formation assay

Logarithmic phase cell suspensions (50 cells/mL) were inoculated into a six-well plate (2 mL per well) and incubated for 10–14 d. The cells were then fixed in ice-cold methanol for 15 min and stained with a 0.1% crystal violet solution (Solarbio). Images of each cell were captured using a camera.

### Fe² ⁺ detection

Cells were placed in 1.5 mL Eppendorf tubes, sonicated, and centrifuged to collect the supernatant. Fe² ⁺ content was detected in the supernatant using the Fe² ⁺ detection kit (Solarbio) according to the manufacturer’s instructions.

### Reactive oxygen species (ROS) detection

ROS levels were detected using a ROS detection kit (Solarbio) according to the manufacturer’s instructions. After removing the culture medium, 1 mL of DCFH-DA dilution solution was added to the cells and incubated for 45 min. Cells were washed with serum-free medium and observed and photographed using a DMI8 fluorescence microscope (Leica, Wetzlar, Germany).

### GSH and NADPH content assays

Cells were collected into 1.5 mL Eppendorf tubes, sonicated, and centrifuged to collect the supernatant. The GSH and NADPH levels in the supernatant were determined using the GSH Assay Kit (KeyGEN Biotech, Nanjing, China) and NADP + /NADPH Assay Kit (Abcam, Cambridge, MA, USA), respectively.

### Immunofluorescence staining

Cells were fixed with 4% paraformaldehyde (Solarbio) for 10 min and then treated with 0.1% Triton X-100 (KeyGEN BioTECH) for 20 min. The cells were then blocked with 3% BSA and incubated overnight at 4 °C with the TRIB3 antibody (1:100 dilution). The following day, the primary antibody was conjugated to the corresponding fluorescent secondary antibody, and the cell nuclei were stained with DAPI staining solution (0.5 μg/mL, Solarbio). Observations and photographs were taken using a DMI8 fluorescence microscope.

### Flow cytometry

Cells (4 × 10⁶ cells/mL) were collected in a 1.5 mL Eppendorf tube. Propidium iodide (PI) solution with a working concentration of 10 μg/mL (5 μL of 1 mg/mL PI stock solution) and 400 μL of 1 × binding buffer were added and mixed well. Apoptosis was detected using the Calibur C6 flow cytometer (BD Biosciences, San Jose, CA, USA).

### Animal experiments

This study was approved by the Ethics Committee of the Fourth Hospital of Hebei Medical University (IACUC-4th Hos Hebmu-20250565) and adhered to the ARRIVE guidelines. All surgery was performed under isoflurane anesthesia, and all efforts were made to minimize suffering. Male BALB/c nude mice aged 5–6 weeks were purchased from Chengdu Dossy Experimental Animals Co., Ltd. (Chengdu, China). Nude mice were subcutaneously inoculated with either 5 × 10⁶ MKN-45 vector or MKN-45 OE-PTPRE cells into the right anterior dorsal region. Grouping was performed when the tumour size reached 120 mm^3^. MKN-45 Vector tumour-bearing mice were randomly divided into Control and 5-FU groups, and MKN-45 OE-PTPRE tumour-bearing mice into Control, 5-FU, and 5-FU + erastin groups (n = 4 per group). 5-FU (25 mg/kg) was administered intraperitoneally (i.p.) 3 × /week, whereas erastin (20 mg/kg) was administered i.p. 1 × /week. The control group received the corresponding vehicle. Monitor the laboratory animals daily. Measure body weight and tumour volume three times a week for each group. On day 21, mice were euthanized using isopentane and carbon dioxide, after which tumor tissues were harvested and weighed. This will be considered a humane endpoint when animals exhibit unrelievable suffering, experience a weight loss of ≥15% within one week, develop a tumor volume of ≥2000 mm^3^ (or accounting for ≥10% of the animal’s body weight), or are near death.

### Immunohistochemical staining

Tissue sections (4 µm) were prepared from the fixed tumour sections. Antigen retrieval: deparaffinized/rehydrated sections in 10 mM citrate buffer (pH 6.0, ZSGB-BIO, Beijing, China), heated at 121°C for 15 min (pressure cooker), cooled and rinsed 3× with PBST (5 min each). IHC staining (ZSGB-BIO kit, per instructions): 3% H₂O₂ blocking (10 min), 5% BSA blocking (30 min, 37°C), PTPRE antibody incubation (1:50, 4°C overnight). After PBST washes, secondary antibody (HRP-conjugated, 30 min, 37°C), DAB visualization, hematoxylin counterstaining, dehydration, mounting and image acquisition via upright microscope.

### Statistical analysis

Statistical analysis was performed using SPSS 22.0 and GraphPad Prism 8.0. Experimental data are expressed as mean ± standard deviation (SD). Data comparisons among multiple groups were performed using one-way analysis of variance, whereas pairwise multiple comparisons were conducted using least significant difference t-tests. Differences were considered statistically significant at *P* < 0.05. Full-length uncropped western blot images for all immunoblot experiments are provided in S1 File. All raw quantitative data used for statistical calculation and graph plotting are available in S2 File.

## Results

### PTPRE induces resistance to 5-FU in gastric cancer cells

**Fig 1:**
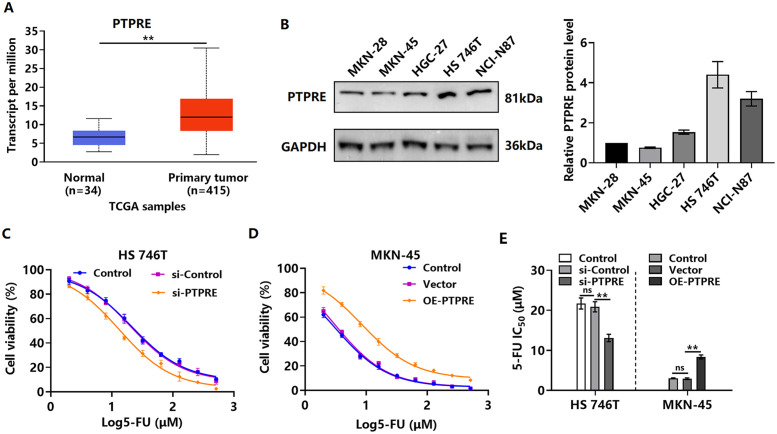
Effect of PTPRE on gastric cancer cell resistance to 5-FU. **(A)** Comparison of PTPRE expression between gastric cancer and normal gastric tissue samples in the TCGA database. **(B)** PTPRE expression levels in five gastric cancer cell lines. Relative PTPRE protein expression levels were normalized to MKN-28 cells. n = 3. **(C)** Changes in sensitivity to 5-FU in HS 746T cells after PTPRE knockdown. n = 3. **(D)** Changes in sensitivity to 5-FU in MKN-45 cells after PTPRE overexpression. n = 3. **(E)** IC50 values of 5-FU for each cell group. n = 3. Experimental data are shown as mean ± SD and representative of three independent experiments. ^**^ *P* ＜ 0.01.

Analysis of the TCGA database revealed that PTPRE expression was upregulated in gastric cancer tissues (Fig 1A). To select an appropriate cell line for our study, we examined PTPRE expression levels in several commonly used gastric cancer cell lines. Among them, HS 746T exhibited high PTPRE expression, whereas MKN-45 exhibited relatively low PTPRE expression (Fig 1B). To validate the role of PTPRE in gastric cancer cell resistance to 5-FU, exogenous PTPRE regulation was performed in HS 746T and MKN-45 cells. The MTT assay results showed that the downregulation of PTPRE protein expression enhanced the sensitivity of HS 746T cells to 5-FU (Figs 1C and 1D). In contrast, PTPRE overexpression reduced the sensitivity of MKN-45 cells to 5-FU (Figs 1C and 1D).

### PTPRE inhibits ferroptosis in gastric cancer cells

**Fig 2:**
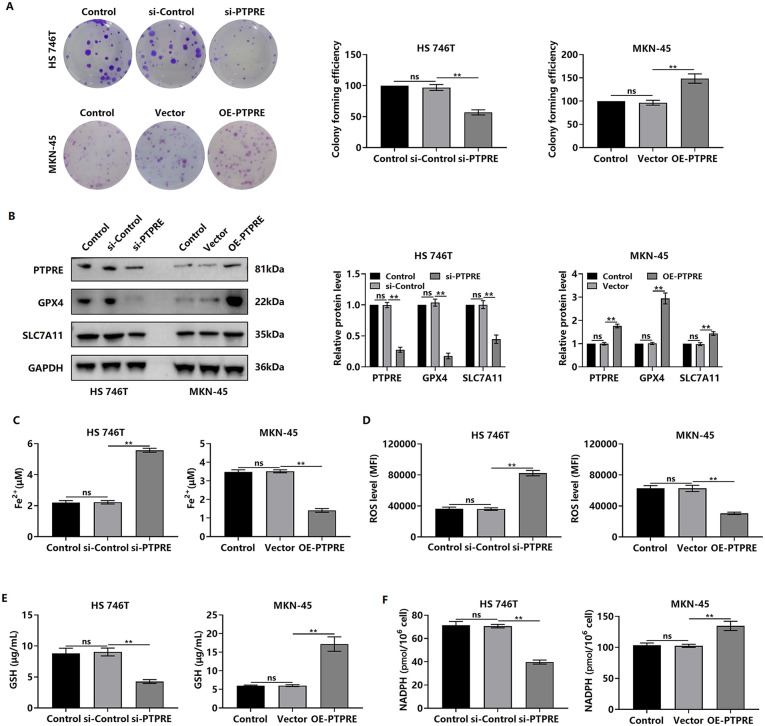
Effects of PTPRE on ferroptosis in gastric cancer cells. **(A)** Colony formation capacity, **(B)** GPX4 and SLC7A11 protein expression in each group of cells. n = 3.

To investigate the potential mechanism by which PTPRE induces 5-FU resistance in gastric cancer cells, we examined the effects of PTPRE on ferroptosis. Plate cloning assays demonstrated that the downregulation of PTPRE protein expression reduced colony formation in HS 746T cells. In contrast, PTPRE overexpression enhanced colony formation in MKN-45 cells (Fig 2A). The indicators of iron metabolism and antioxidant system abnormalities induced by ferroptosis were simultaneously examined. PTPRE downregulation led to the depletion of GPX4, SLC7A11, GSH, and NADPH in HS 746T cells, accompanied by the accumulation of Fe²⁺ and ROS (Figs 2B–F). Following the exogenous upregulation of PTPRE expression, MKN-45 cells exhibited results opposite to those observed in HS 746T cells (Figs 2B–F).

Data were normalized to the Control group. **(C)** Fe² ⁺ levels, **(D)** ROS levels, **(E)** GSH levels, and **(F)** NADPH levels in each group of cells. n = 3. Experimental data are shown as mean ± SD and representative of three independent experiments. ^**^P ＜ 0.01.

### PTPRE inhibits ferroptosis in gastric cancer cells by upregulating TRIB3

**Fig 3:**
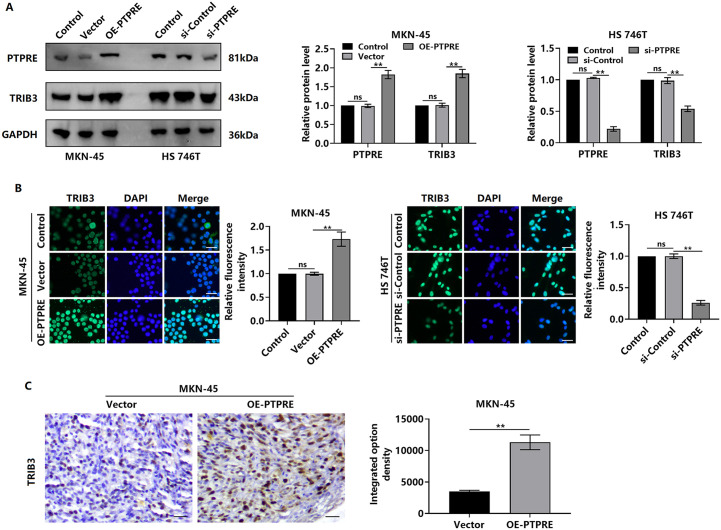
Effects of PTPRE on TRIB3 protein expression in gastric cancer cells. **(A)** Western blot analysis revealed TRIB3 protein expression after exogenous regulation of PTPRE protein expression in MKN-45 and HS 746T cells. Relative protein expression levels were normalized to the Control group. n = 3. **(B)** Immunofluorescence analysis of TRIB3 expression after exogenous regulation of PTPRE protein expression in MKN-45 and HS 746T cells. Scale bar = 30 µm. Relative fluorescence intensity was normalized to the control group. n = 3. **(C)** Changes in TRIB3 protein expression in PTPRE-overexpressing xenograft tumours. n = 3. Scale bar = 20 µm. Experimental data are shown as mean ± SD and representative of three independent experiments. ^**^P ＜ 0.01.

**Fig 4:**
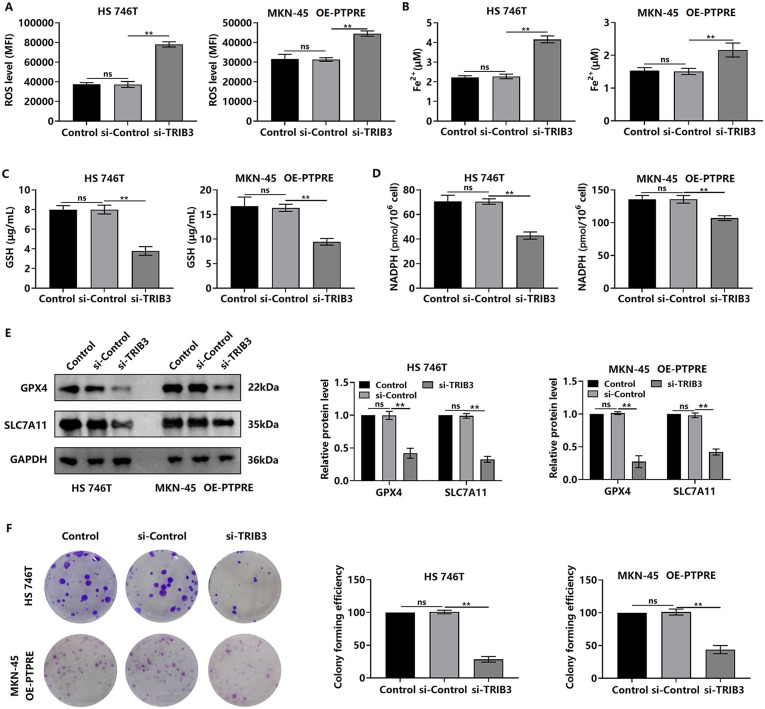
Role of TRIB3 in PTPRE-mediated ferroptosis suppression in gastric cancer cells. Changes in ROS **(A)**, Fe²⁺ **(B)**, GSH **(C)**, and NADPH **(D)** levels in MKN-45 and HS 746T cells after exogenous regulation of PTPRE protein expression. n = 3. **(E)** GPX4 and SLC7A11 protein expression in each group. Relative protein expression levels were normalized to the Control group. n = 3. **(F)** Colony formation capacity of the cells in each group. Data were normalized to the Control group. n = 3. Experimental data are shown as mean ± SD and representative of three independent experiments. ^**^P ＜ 0.01.

We further investigated the signalling mechanisms whereby PTPRE inhibits ferroptosis in gastric cancer cells. PTPRE overexpression upregulated TRIB3 protein expression in MKN-45 cells (Figs 3A and 3B). PTPRE downregulation induced a decrease in TRIB3 protein expression in HS 746T cells (Figs 3A and 3B). TRIB3 protein expression was also elevated in MKN-45 xenograft tumours overexpressing TRIB3 (Fig 3C). We assessed the expression of ferroptosis-related markers to validate the role of TRIB3 in suppressing ferroptosis in gastric cancer cells. TRIB3 protein expression reduced GSH, NADPH, GPX4, and SLC7A11 levels in HS 746T and MKN-45 OE-PTPRE cells and promoted Fe²⁺ and ROS accumulation (Figs 4A–E). Furthermore, interference with TRIB3 protein expression suppressed the colony formation capacity of HS 746T and MKN-45 OE-PTPRE cells (Fig 4F).

### Src/FAK signalling pathway is critical for PTPRE-mediated upregulation of TRIB3

**Fig 5:**
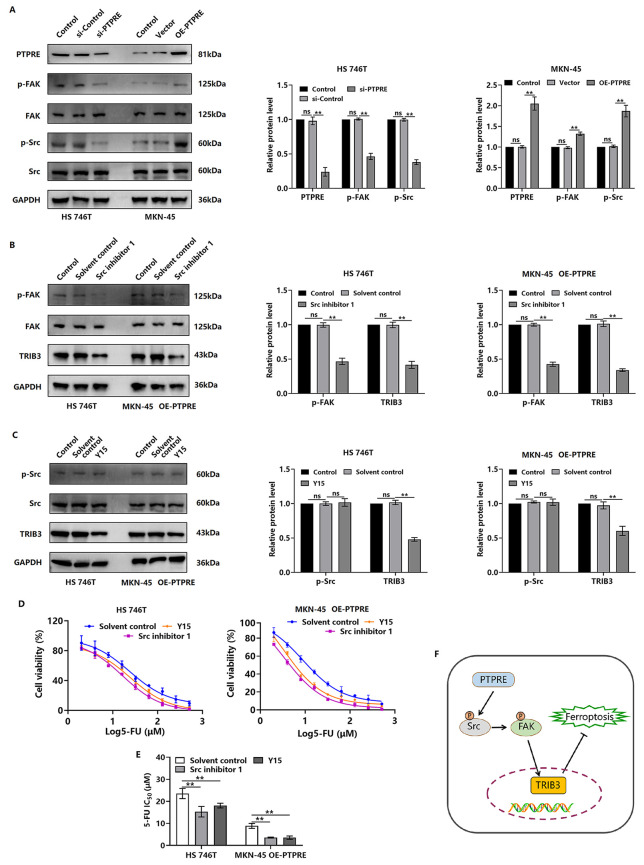
Role of the Src/FAK signalling pathway in PTPRE-mediated TRIB3 upregulation. **(A)** Src and FAK phosphorylation levels in MKN-45 and HS 746T cells following exogenous regulation of PTPRE protein expression. n = 3. **(B)** Effects of Src inhibitor 1 on PTPRE-induced upregulation of p-FAK and TRIB3 protein expression. n = 3. **(C)** Effects of a FAK inhibitor (Y15) on the PTPRE-induced upregulation of p-Src and TRIB3 protein expression. n = 3. **(D)** The effect of Src or FAK inhibition on 5-FU-induced proliferation suppression in HS 746T and MKN-45 OE-PTPRE cells. n = 3. **(E)** IC50 values of 5-FU for each cell group. n = 3. **(F)** Schematic diagram of the signaling pathway by which PTPRE inhibits ferroptosis through the Src/FAK/TRIB3 axis. Relative protein expression levels were normalized to the Control group. Experimental data are shown as mean ± SD and representative of three independent experiments. ^**^P ＜ 0.01.

Our subsequent studies revealed that interference with PTPRE protein expression downregulated Src and FAK phosphorylation in HS 746T cells. Conversely, Src and FAK phosphorylation levels were elevated in PTPRE overexpressing MKN-45 cells (Fig 5A). The inhibitory effects of Src and FAK were examined separately to investigate their roles in the upregulation of TRIB3 expression in PTPRE cells. Both Src inhibitors 1 and Y15 suppressed TRIB3 protein expression in HS 746T and MKN-45 OE-PTPRE cells (Figs 5B and 5C). Src inhibitor 1 suppressed FAK phosphorylation in HS 746T and MKN-45 OE-PTPRE cells (Fig 5B); however, Y15 did not affect Src phosphorylation levels (Fig 5C). In addition, treatment with Src inhibitor 1 or Y15 enhances the sensitivity of HS 746T and MKN-45 OE-PTPRE cells to 5-FU (Figs 5D and 5E). These findings indicate that PTPRE enhances the resistance of gastric cancer cells to 5-FU by inhibiting ferroptosis via the Src/FAK/TRIB3 pathway (Fig 5F).

### Ferroptosis inhibition by PTPRE/TRIB3 reduced the 5-FU sensitivity in gastric cancer cells

**Fig 6:**
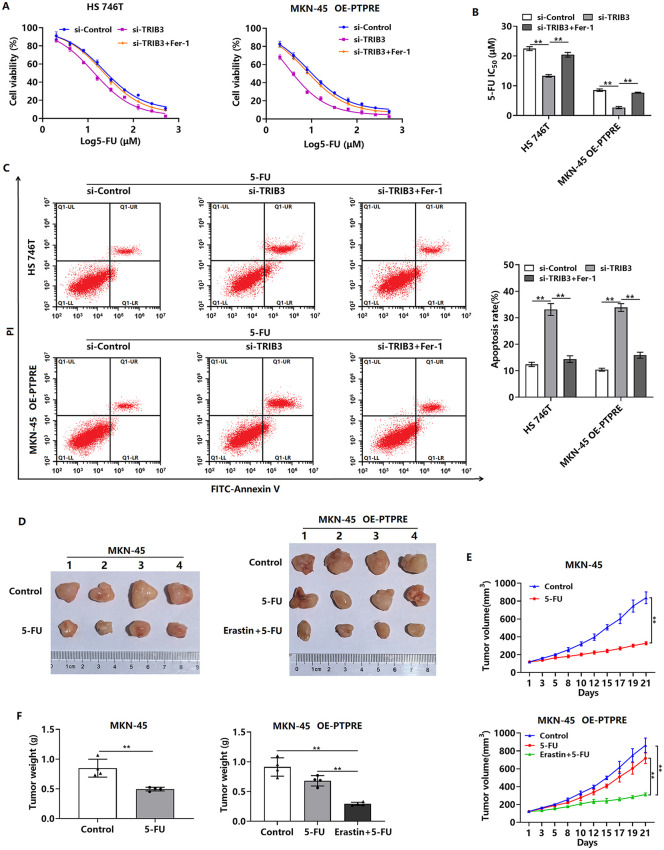
PTPRE/TRIB3 inhibition of ferroptosis reduces the sensitivity of gastric cancer cells to 5-FU. **(A)** Effects of TRIB3 silencing and/or inhibiting ferroptosis on 5-FU-induced proliferation inhibition in HS 746T and MKN-45 OE-PTPRE cells. n = 3. **(B)** IC50 values of 5-FU for each cell group. n = 3. **(C)** Effects of silencing TRIB3 and/or inhibiting ferroptosis on 5-FU-induced apoptosis in HS 746T and MKN-45 OE-PTPRE cells. n = 3. **(D)** Photograph of MKN-45 and MKN-45 OE-PTPRE xenograft tumours. n = 4. **(E)** Tumour volume in each group. n = 4. **(F)** Tumour weight in each group. n = 4. Experimental data are shown as mean ± SD and representative of three independent experiments. ^**^P ＜ 0.01.

Lastly, we examined the relationship between PTPRE/TRIB3-mediated ferroptosis and resistance of gastric cancer cells to 5-FU, demonstrating that knockdown of TRIB3 enhanced the sensitivity of HS 746T and MKN-45 OE-PTPRE cells to 5-FU (Figs 6A–C). Fer-1 can reverse the above observed effects induced by TRIB3 downregulation (Figs 6A–C). Additionally, xenograft experiments demonstrated that, compared to MKN-45 tumours, the therapeutic efficacy of 5-FU was reduced in MKN-45 OE-PTPRE xenografts (Figs 6D–F), whereas erastin (a specific ferroptosis inducer) treatment enhanced the sensitivity of MKN-45 OE-PTPRE xenografts to 5-FU (Figs 6D–F).

## Discussion

PTPRE is involved in tumorigenesis and the progression of malignant tumours, such as breast, liver, and thyroid cancers. Sustained activation of the EGFR-mediated ERK1/2 pathway induces PTPRE expression and activation, thereby maintaining the survival of breast cancer cells [19]. PTPRE is upregulated in acute myeloid leukaemia [20], renal cell carcinoma [21], and hepatocellular carcinoma [16]. PTPRE is also overexpressed in thyroid cancer and is correlated with a poor prognosis, as it induces thyroid cancer cell migration, invasion, and epithelial-mesenchymal transition by activating AKT and ERK1/2 [14]. Recent studies have linked PTPRE to anticancer drug therapy resistance, as it regulates etoposide resistance in retinoblastomas through SGK3, AKT, and ERK1/2 [17]. Studies have also reported that PTPRE plays a crucial role in sorafenib resistance in hepatocellular carcinoma, and that inhibiting PTPRE enhances therapeutic efficacy by suppressing the expression of MYC and its target genes [18]. However, the relationship between PTPRE expression and drug resistance in gastric cancer remains unclear. Our findings demonstrated that PTPRE is overexpressed in gastric cancer tissues and is correlated with 5-FU resistance.

Our further research has shown that PTPRE can inhibit ferroptosis in gastric cancer cells in vitro. Ferroptosis, a novel form of cell death, is a promising therapeutic strategy for cancer treatment [22]. Phosphoglycerate dehydrogenase suppresses ferroptosis and promotes malignant progression in bladder cancer by interacting with poly(rC)-binding protein 2 to upregulate SLC7A11 expression [23]. Furthermore, targeted induction of ferroptosis inhibits tumour cell proliferation. Sulfadiazine inhibits the proliferation of oesophageal cancer cells by inducing ferroptosis [24]. Ketamine suppresses the proliferation of breast cancer cells by targeting the KAT5/GPX4 axis, inducing ferroptosis, and promoting apoptosis [25]. Ferroptosis has also been implicated in tumour cell invasion, stemness, and angiogenesis. NADPH oxidase 4 enhances ferroptosis by increasing ROS levels, thereby reducing the invasive capacity of gastric cancer cells [26]. Ursodeoxycholic acid suppresses the stemness and proliferation of triple-negative breast cancer cells via nuclear factor erythroid-related factor 2-mediated ferroptosis [27]. Piceatannol induces ferroptosis in prostate cancer cells by interfering with HIF1A histone palmitoylation, thereby inhibiting Sema3A-mediated angiogenesis and PD-L1 expression [28]. Ferroptosis regulation is also linked to the sensitivity to immunotherapy and chemotherapy. TMEM16A suppresses ferroptosis in cancer cells via the PI3K/Akt signalling pathway, thereby inducing immunotherapy resistance through a series of immunomodulatory effects [29]. Lung cancer research has shown that targeting carnitine palmitoyltransferase 1A can induce ferroptosis and enhance the efficacy of immunotherapy [30]. Ferroptosis also contributes to endocrine therapy resistance. RelB activates GPX4 to suppress apoptosis and induces tamoxifen resistance in breast cancer [31]. Ouyang et al. [32] reported that activating transcription factor 3 enhances cisplatin sensitivity in gastric cancer cells by inducing ferroptosis by blocking the Nrf2/Keap1/xCT signalling pathway. Therefore, ferroptosis inhibition may exert antitumour effects. Studies have shown that promoting ROS-mediated ferroptosis can enhance the efficacy of 5-fluorouracil treatment in gastric cancer [33]. These findings suggest that, in this study, PTPRE contributed to 5-FU resistance by inhibiting ferroptosis in gastric cancer cells.

TRIB3 is a key factor in tumorigenesis and cancer progression and is essential for lipid metabolism, cell differentiation, and cell survival [34,35]. Recent studies suggest that TRIB3 promotes the progression of head and neck squamous cell carcinoma by inhibiting ferroptosis [36]. Palbociclib downregulates TRIB3 expression by inhibiting the SOX2/SLC7A11 pathway, thereby promoting ferroptosis in prostate cancer cells [37]. Methyltransferase-like 14 inhibits TRIB3 via an m6A-YTHDF2-dependent pathway, thereby promoting ferroptosis in colorectal cancer cells [38]. Studies have shown that the downregulation of TRIB3-induced ferroptosis reduces sunitinib resistance in clear cell renal cell carcinoma [39]. These findings suggest that TRIB3 is a key inhibitor of tumour ferroptosis and may represent a potential therapeutic target. Therefore, we investigated the role of TRIB3 in the PTPRE-mediated suppression of ferroptosis and 5-FU resistance in gastric cancer cells. We found that PTPRE inhibited ferroptosis in gastric cancer cells by upregulating TRIB3 expression, and this effect correlated with 5-FU resistance. Thus, PTPRE causes 5-FU resistance by inhibiting ferroptosis in gastric cancer cells via TRIB3. Therefore, inducing ferroptosis may be a potential strategy for overcoming 5-FU resistance in gastric cancer cells. However, to date, no ferroptosis inducers have been approved as anticancer drugs, which thus opens up broad research opportunities and clinical translation potential for exploring the role of ferroptosis-related regulatory targets (such as PTPRE) in reversing 5-FU resistance in gastric cancer. Previous studies have reported that Src is a downstream signalling target for the biological effects of PTPRE [40]. Therefore, we investigated the relationship between PTPRE-mediated upregulation of TRIB3 protein expression and Src. Our findings revealed that PTPRE induces FAK phosphorylation by activating Src; however, inhibition of the Src/FAK signalling pathway reversed the PTPRE-induced upregulation of TRIB3 protein expression. These findings suggest that PTPRE induces TRIB3 upregulation by activating the Src/FAK signalling pathway.

In summary, PTPRE promotes 5-FU resistance in gastric cancer cells by inhibiting ferroptosis through the Src/FAK/TRIB3 axis. Targeting PTPRE and its downstream signalling pathways may represent a potential therapeutic strategy for overcoming chemotherapy resistance in gastric cancer.

## Supporting information

S1 File Raw images. (PDF)

S2 File Minimal data set. (PDF)
