# Supplementary material for: PTPRE promotes gastric cancer cell resistance to 5-fluorouracil by inhibiting ferroptosis via the Src/FAK/TRIB3 axis
Source: PLoS One. 2026 Jun 18;21(6):e0351846. doi: 10.1371/journal.pone.0351846 (PMC13278412; doi:10.1371/journal.pone.0351846)

Fig.1B PTPRE

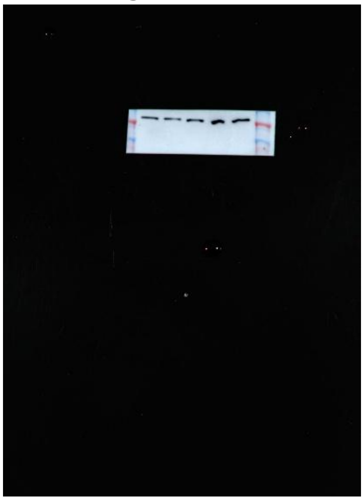

Fig.1B GAPDH

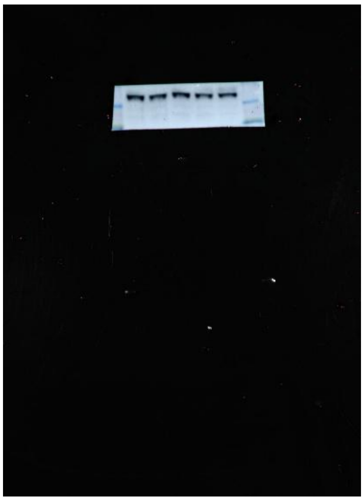

Fig.2B PTPRE

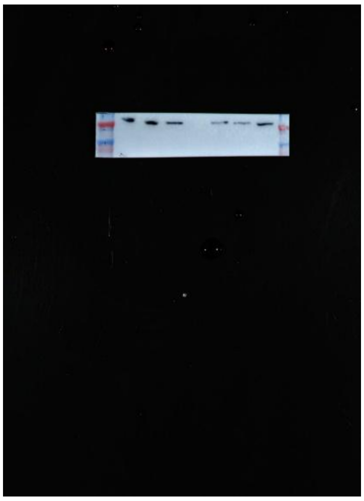

Fig.2B PGPX4

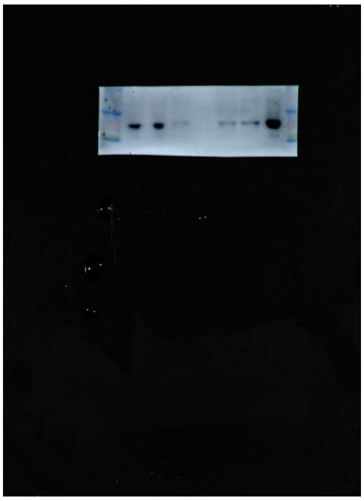

Fig.2B SLC7A11

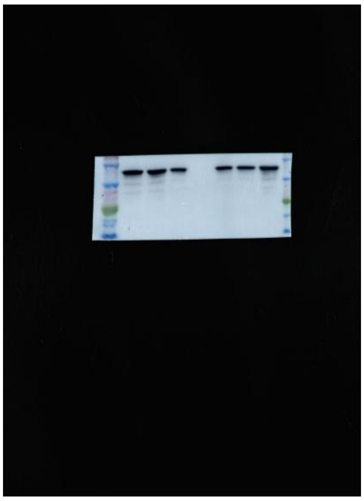

Fig.2B GAPDH

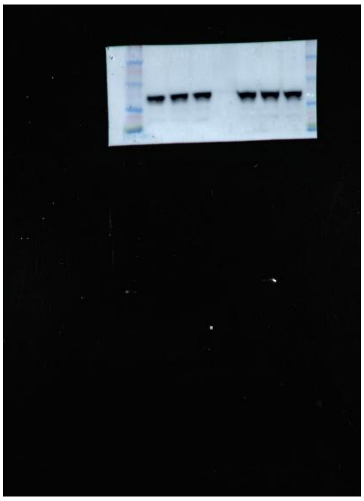

Fig.3A PTPRE

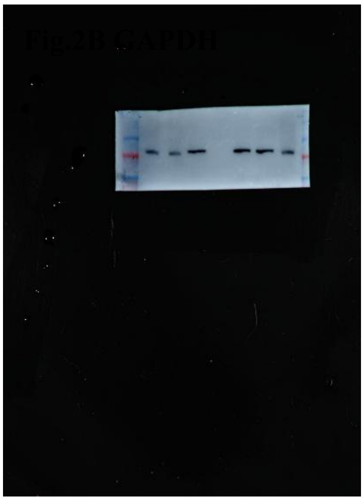

Fig.3A TRIB3

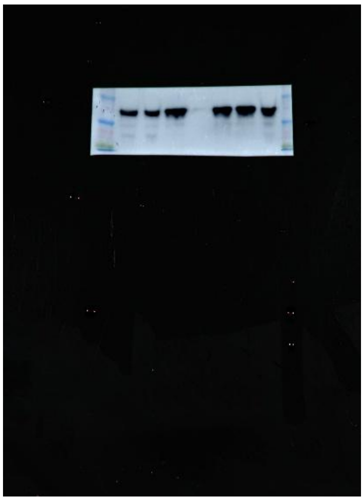

Fig.3A GAPDH

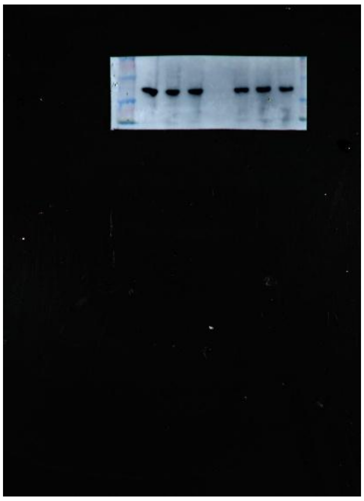

Fig.4E GPX4

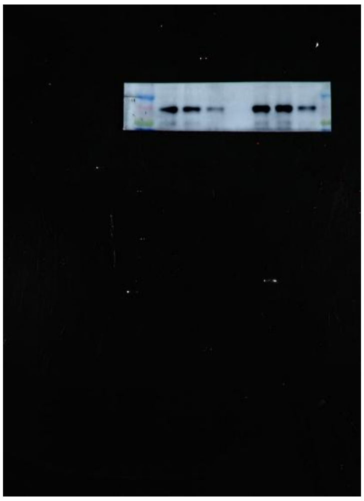

Fig.4E SLC7A11

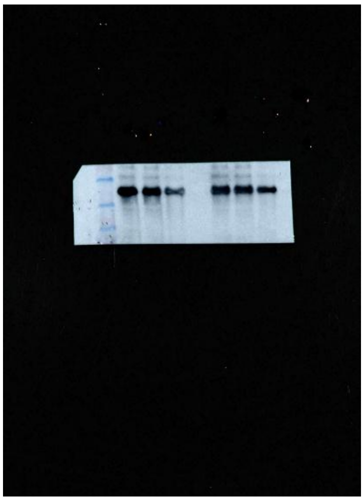

Fig.4E GAPDH

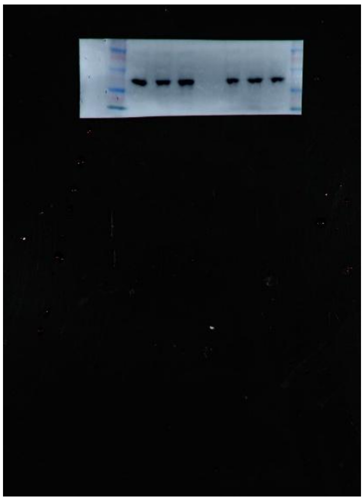

Fig.5A PTPRE

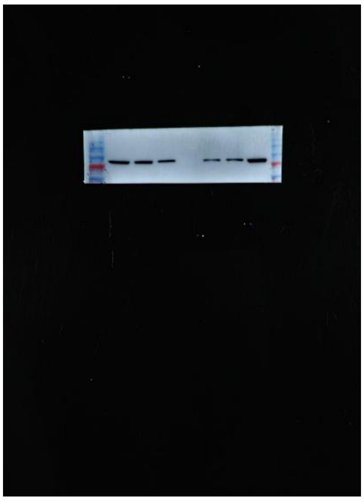

Fig. 5A p-FAK

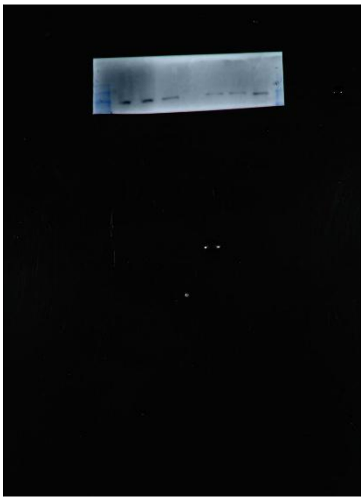

Fig. 5A FAK

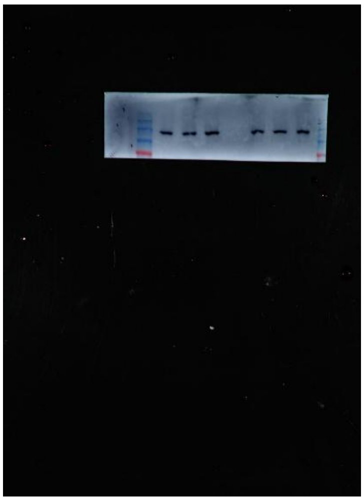

Fig.5A p-Src

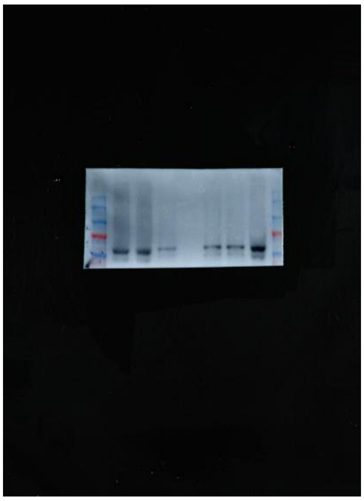

Fig.5A Src

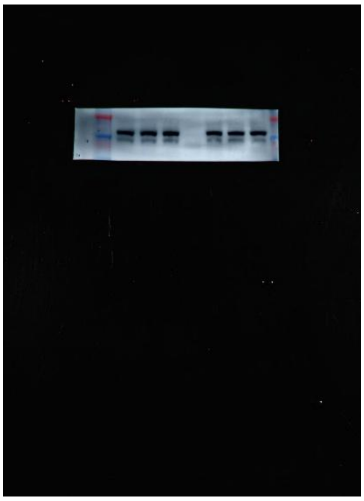

Fig.5A TRIB3

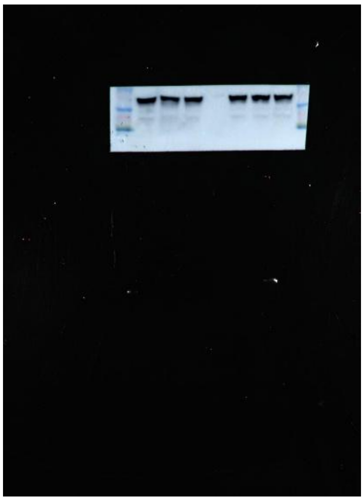

Fig.5B p-FAK

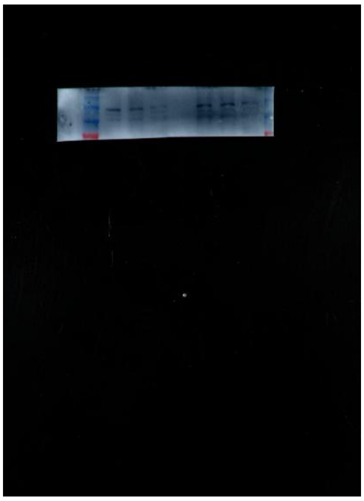

Fig.5B FAK

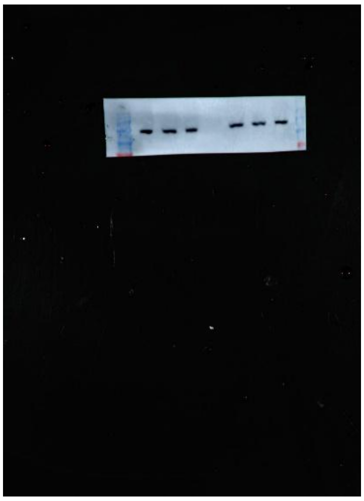

Fig.5B TRIB3

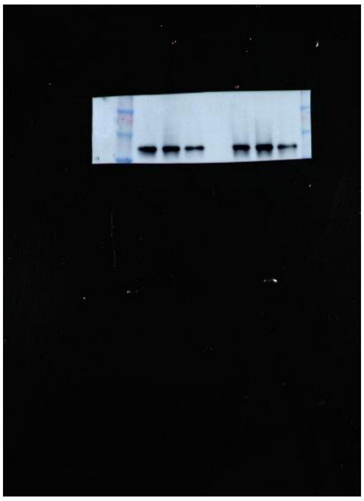

Fig.5B GAPDH

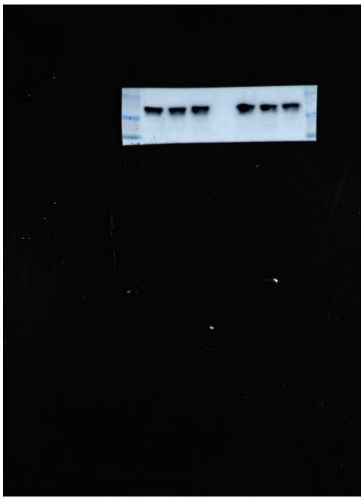

Fig.5C p-Src

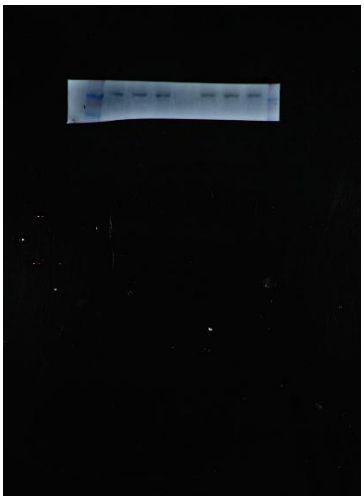

Fig.5C Src

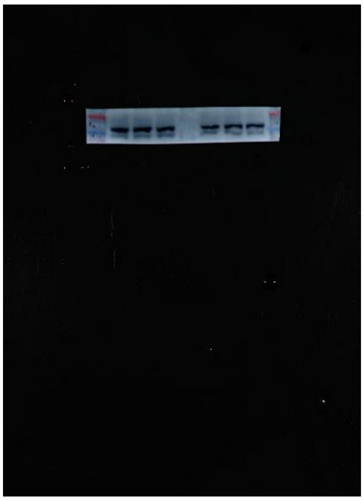

Fig.5C TRIB3

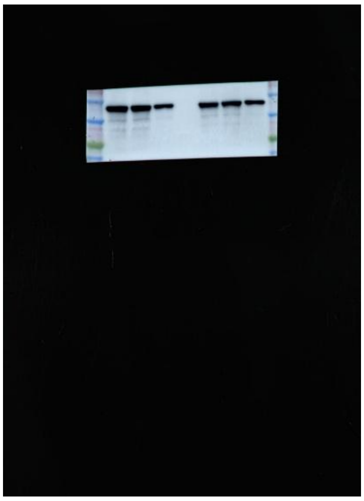

Fig.5C GAPDH

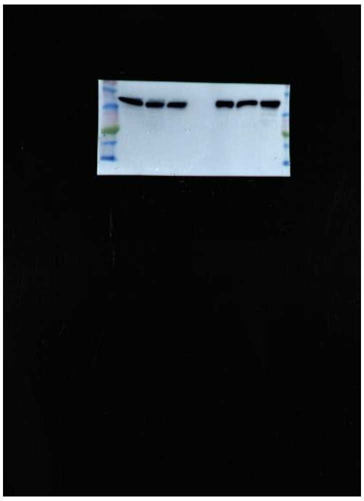

Supplement: S1 File — (PDF) [file pone.0351846.s001.pdf]
