# Supplementary material for: PTPRE promotes gastric cancer cell resistance to 5-fluorouracil by inhibiting ferroptosis via the Src/FAK/TRIB3 axis
Source: PLoS One. 2026 Jun 18;21(6):e0351846. doi: 10.1371/journal.pone.0351846 (PMC13278412; doi:10.1371/journal.pone.0351846)
Supplement: S2 File — (PDF) [file pone.0351846.s002.pdf]

Fig.1A

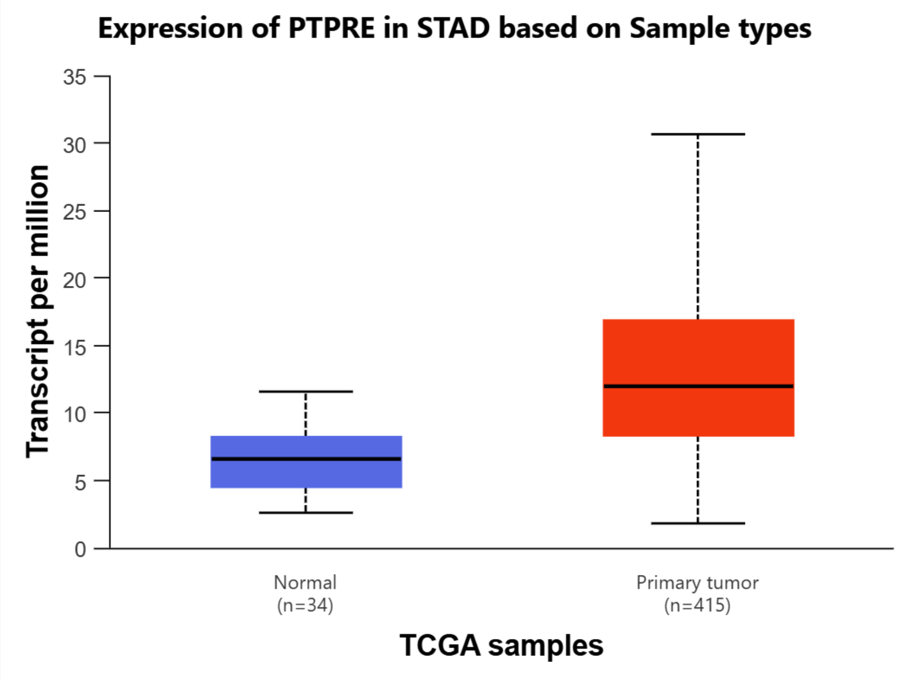

STAD : Stomach adenocarcinomna

Statistics: Unpaired two sample T-test was employed to evaluate levels of significance in expression level between any two sample groups

Comparison	Statistical significance
Normal-vs-Primary	1.62436730732907E-12

Fig.1B PTPRE

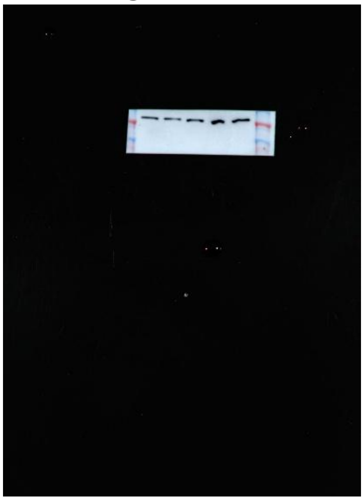

Fig.1B GAPDH

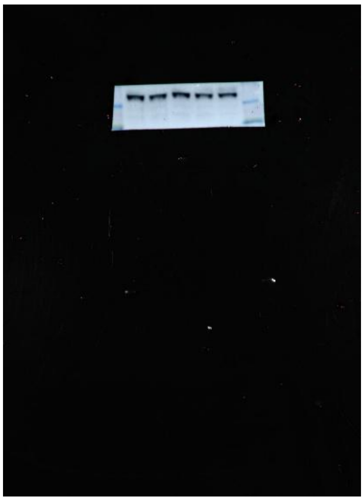

Fig.1C

5-FU (μM)	Control			si-Control			si-PTPRE		
2	90.3	93.2	88.6	90.3	94.6	92.2	88.1	87.2	85.1
4	83.06	80.5	85.1	84.5	82.1	86.6	75.2	77.6	79.3
8	75.0	72.2	77.1	71.2	73.3	74.6	63.3	63.8	59.2
16	60.7	63.5	57.3	58.2	64.3	56.9	42.5	45.6	44.1
32	42.2	45.2	39.1	39.2	43.6	42.1	33.6	32.1	28.7
64	32.1	28.6	31.1	30.5	29.6	30.7	25.6	20.2	23.8
128	25.3	27.5	23.1	23.3	25.1	22	8.2	14.3	15.1
256	17.6	16.4	15.2	19.7	15.2	13.87	8.3	9	6.3
512	12.0	9.2	10.1	11.2	9.6	6.1	2.3	3.5	1.8

Fig.1D

5-FU (μM)	Control			Vector			OE-PTPRE		
2	62.2	59.7	64.5	67.7	65.3	62.1	84.6	82.3	78.6
4	45.3	43.3	46.8	48.2	45.6	49.1	70.3	72.2	68.8
8	28.0	30.2	25.7	26.3	27.2	30.1	56.6	52.2	54.1
16	19.1	22.1	17.4	22.1	23.8	21.1	39.7	42.8	37
32	12.3	10.7	13.6	10.6	10.9	11.5	29.7	26.5	29.3
64	8.1	8.8	7.6	9.2	8.1	10.1	21.2	22.5	19.4
128	6.2	5.1	5.5	8.3	5.2	4.6	16.3	17.2	15.7
256	3.1	3.5	3.2	3.3	3.2	3.2	12.8	13.2	10.6
512	1.0	1.2	2.1	0.8	2.1	1.7	9.3	8.8	7.1

Fig.1E

Control			si-Control			si-PTPRE		
21.07	23.314	20.788	19.59	22.125	20.976	13.22	12.127	13.961

Control			Vector			OE-PTPRE		
3.037	2.893	3.156	2.976	3.097	2.711	8.507	7.832	8.818

Fig.2A

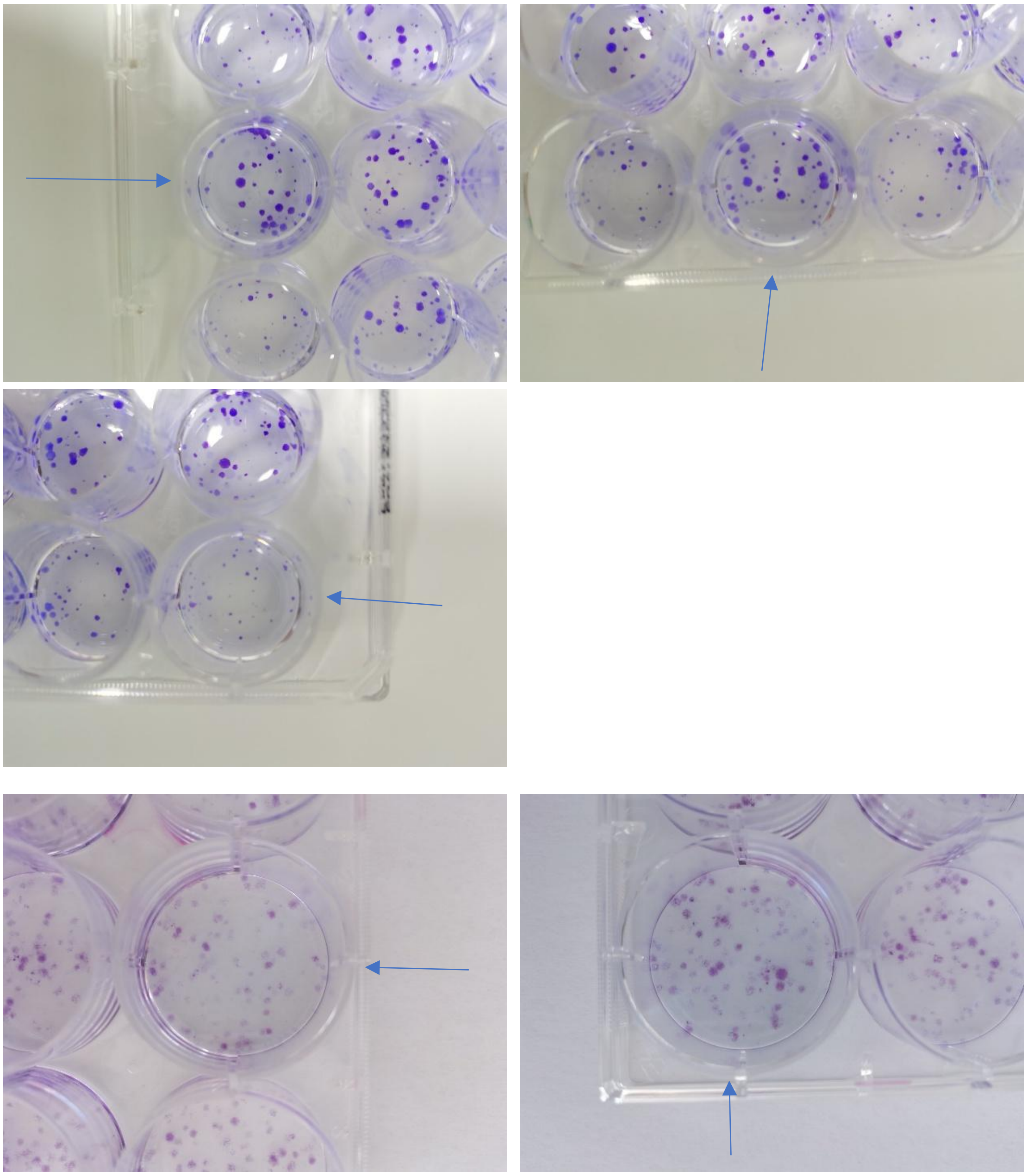

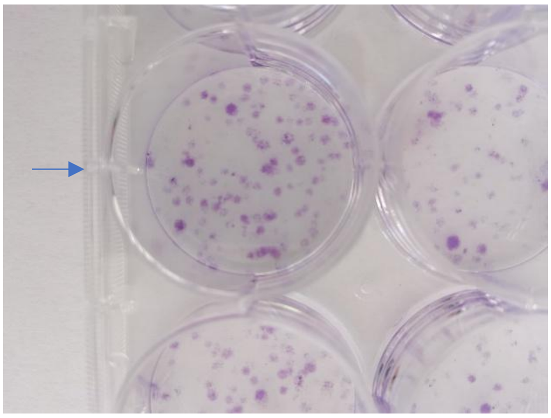

Fig.2B PTPRE

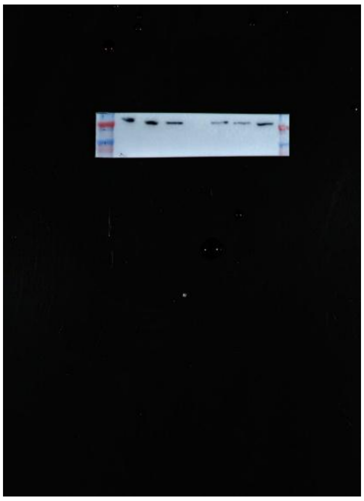

Fig.2B PGPX4

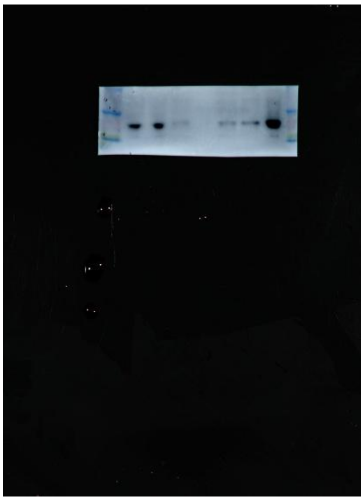

Fig.2B SLC7A11

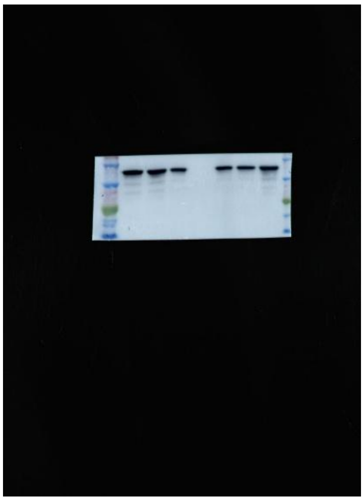

Fig.2B GAPDH

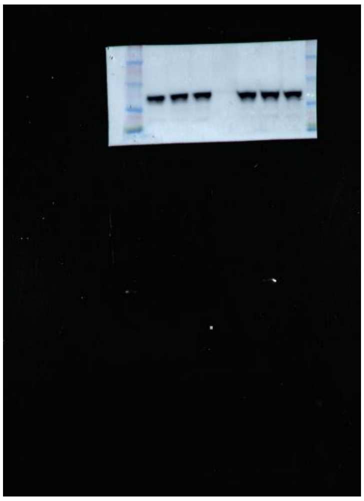

Fig.2C Fe^{2+} (μM)

Control	si-Control	si-PTPRE
2.21	2.09	5.53
2.08	2.27	5.71
2.32	2.29	5.46

Control	Vector	OE-PTPRE
3.61	3.52	1.52
3.43	3.43	1.38
3.38	3.59	1.33

Fig.2D ROS (MFI)

Control	si-Control	si-PTPRE
36261	35915	83691
34196	34162	78314
38472	37537	84716

Control	Vector	OE-PTPRE
63158	64912	30214
58916	57936	28961
65871	64971	32014

Fig.2E GSH (μg/mL)

Control	si-Control	si-PTPRE
8.83	9.02	4.33
7.92	8.39	3.91
9.61	9.66	4.52

Control	Vector	OE-PTPRE
6.02	5.89	17.11
5.78	6.29	15.25
6.13	5.83	19.13

Fig.2F (pM/10^6 cell)

Control	si-Control	si-PTPRE
72.31	70.35	40.35
67.86	69.14	37.76
74.08	72.17	41.01

Control	Vector	OE-PTPRE
103.28	104.61	136.67
107.15	99.62	126.34
100.31	103.07	140.75

Fig.3A PTPRE

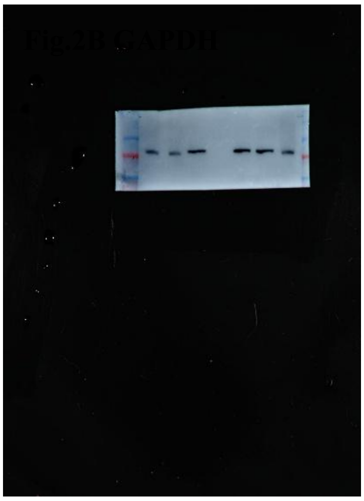

Fig.3A TRIB3

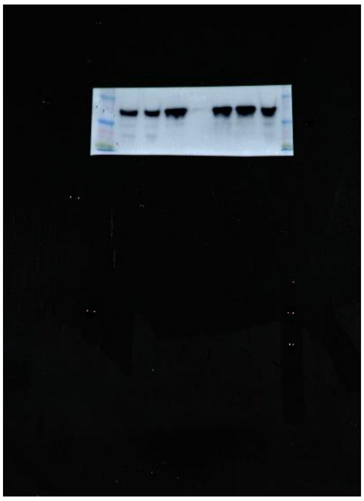

Fig.3A GAPDH

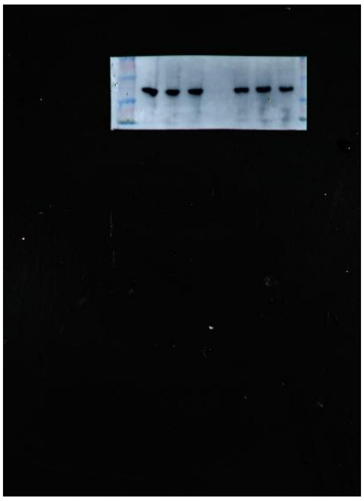

Fig.3B MKN-5

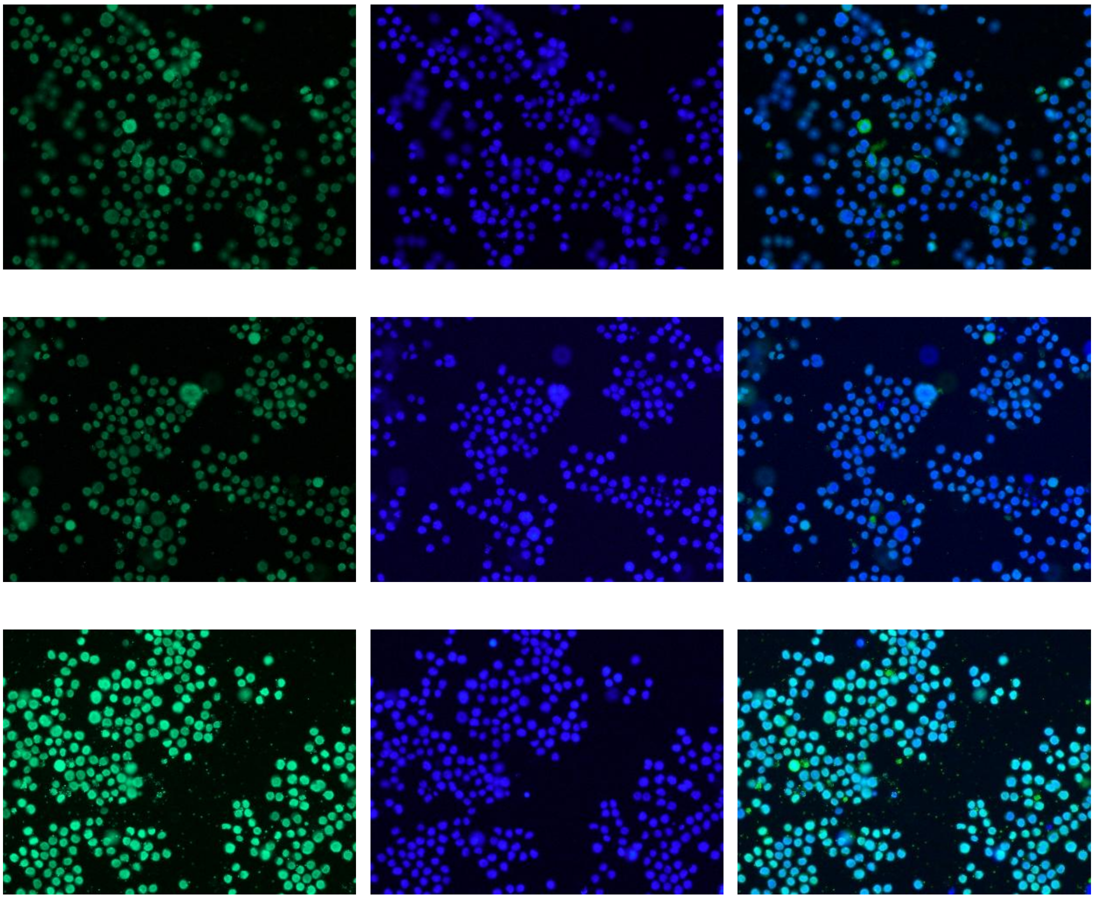

Fig.3B HS 746T

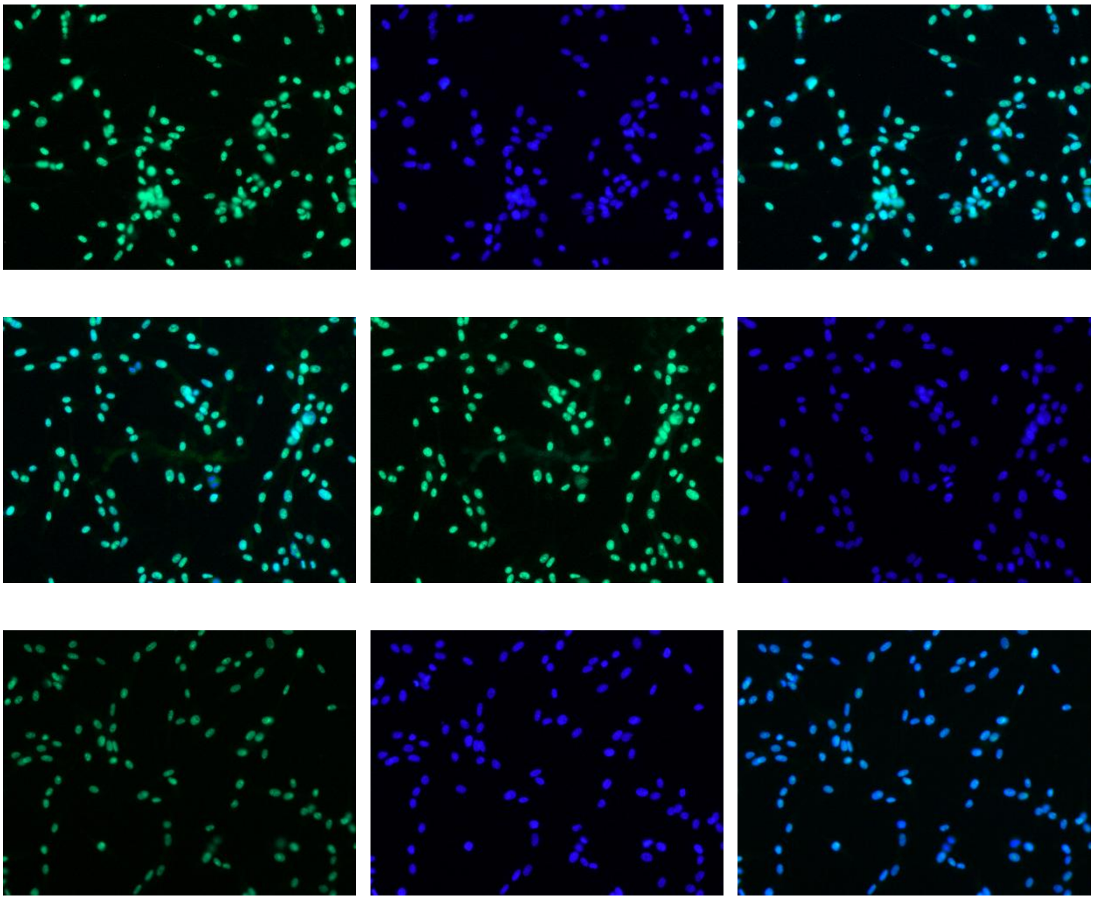

Fig.3C

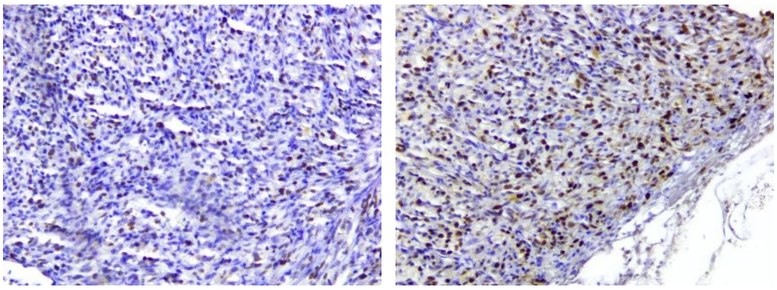

Fig.4A ROS (MFI)

HS746T

Control	si-Control	si-TRIB3
37265	33893	75336
35858	38251	80631
39314	39645	78165

MKN-45 OE-PTPRE

Control	si-Control	si-TRIB3
31666	32365	44363
29136	30589	43285
33925	30982	45967

Fig.4B Fe^{2+} (μM)

HS746T

Control	si-Control	si-TRIB3
2.3	2.14	4.33
2.12	2.35	3.98
2.23	2.33	4.16

MKN-45 OE-PTPRE

Control	si-Control	si-TRIB3
1.63	1.61	2.23
1.45	1.49	1.92
1.51	1.42	2.33

Fig.4C GSH (μg/mL)

HS746T

Control	si-Control	si-TRIB3
8.06	7.82	3.36
7.55	7.66	4.25
8.36	8.51	3.75

MKN-45 OE-PTPRE

Control	si-Control	si-TRIB3
16.23	17.13	9.37
15.15	15.68	10.15
18.72	16.22	8.82

Fig.4D (pM/10^6 cell)

HS746T

Control	si-Control	si-TRIB3
73.65	72.56	45.25
65.17	68.17	39.61
73.49	70.75	43.67

MKN-45 OE-PTPRE

Control	si-Control	si-TRIB3
136.67	139.84	110.52
129.34	128.91	106.92
140.75	137.91	103.14

Fig.4E GPX4

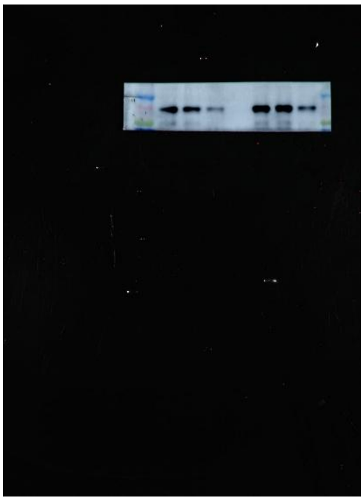

Fig.4E SLC7A11

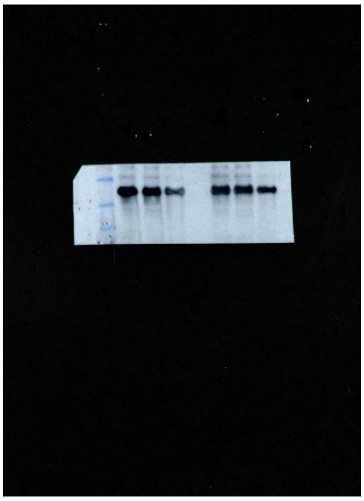

Fig.4E GAPDH

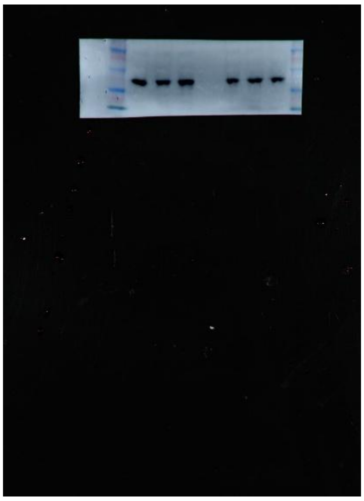

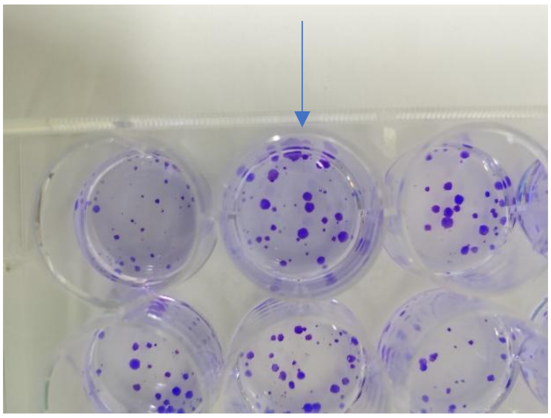
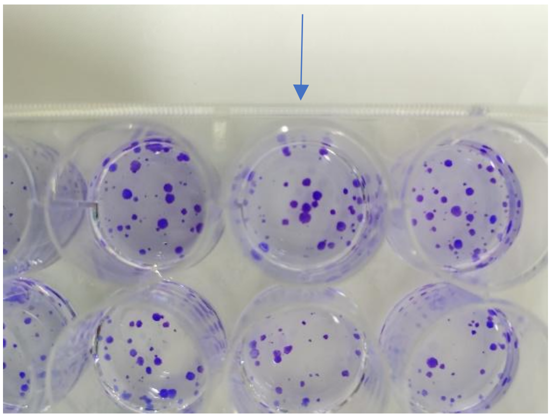
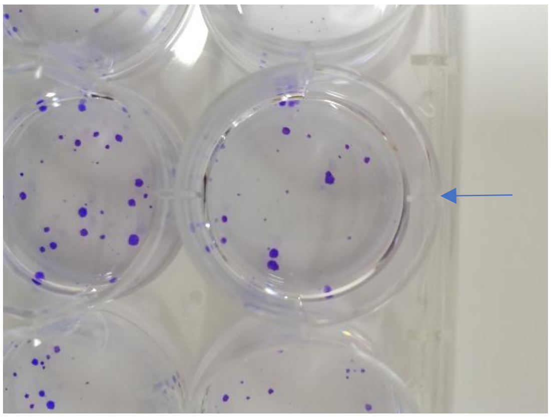
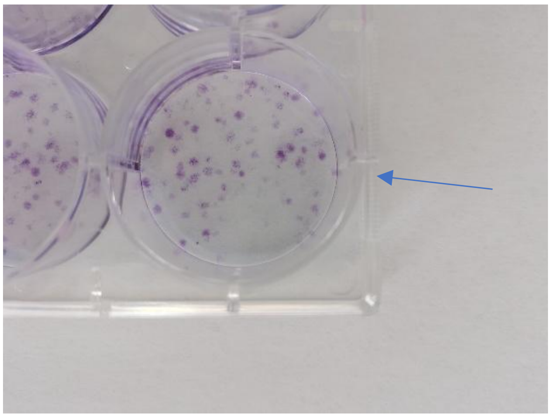
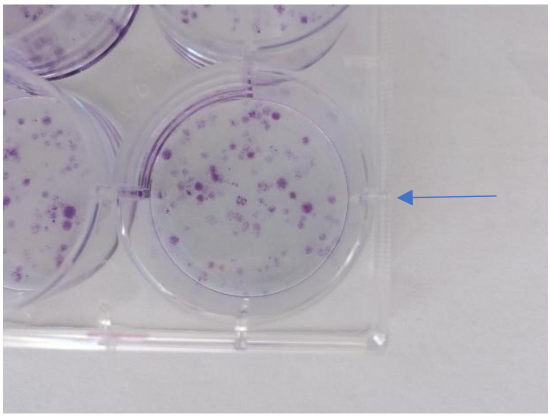
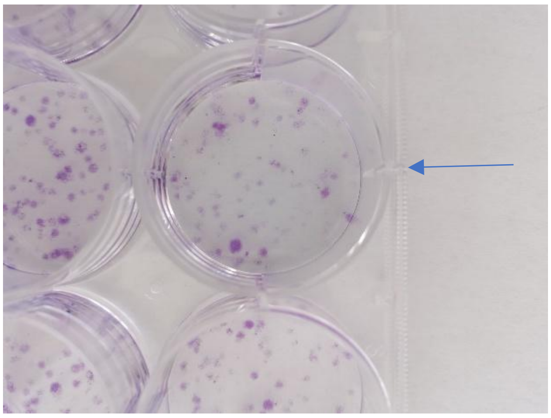

Fig.5A PTPRE

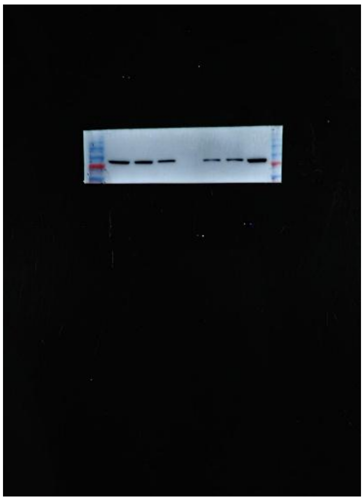

Fig. 5A p-FAK

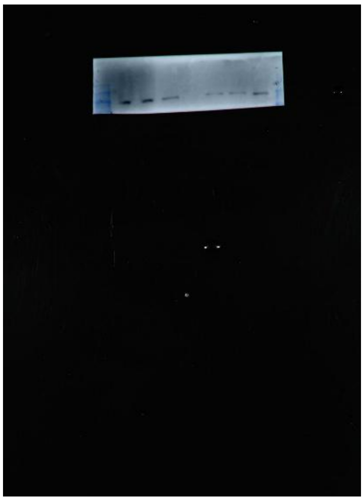

Fig. 5A FAK

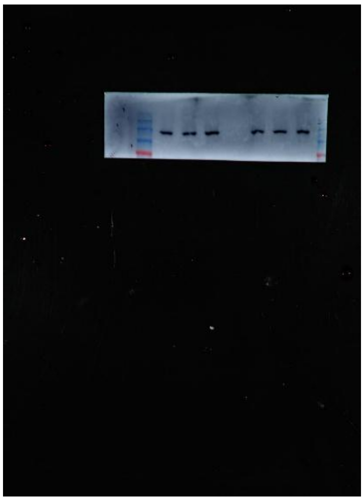

Fig.5A p-Src

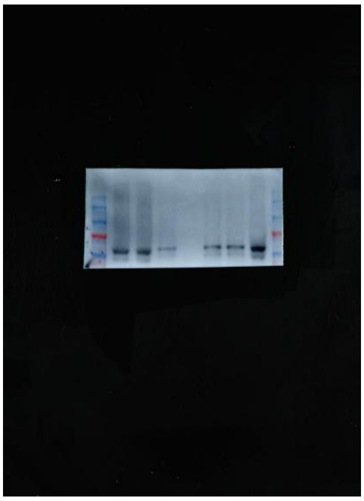

Fig.5A Src

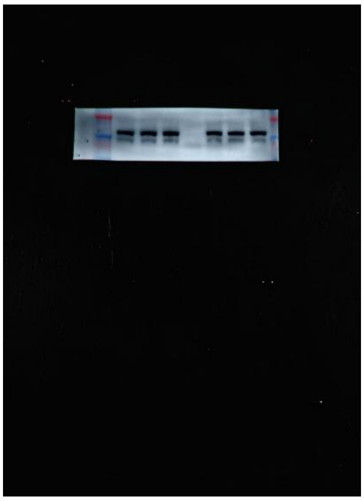

Fig.5A TRIB3

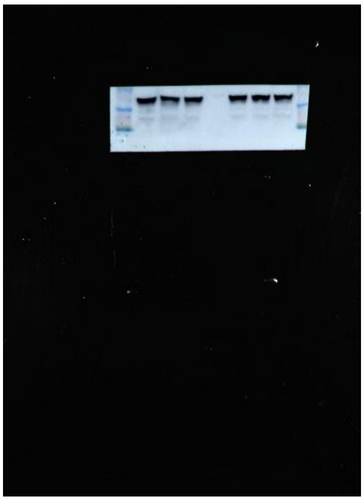

Fig.5B p-FAK

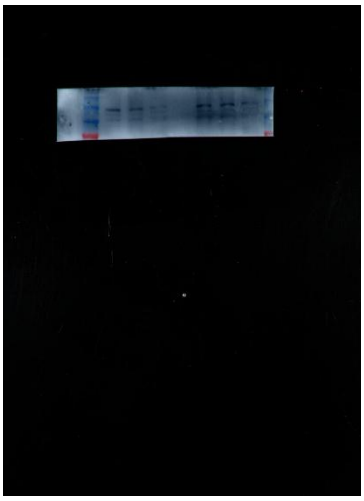

Fig.5B FAK

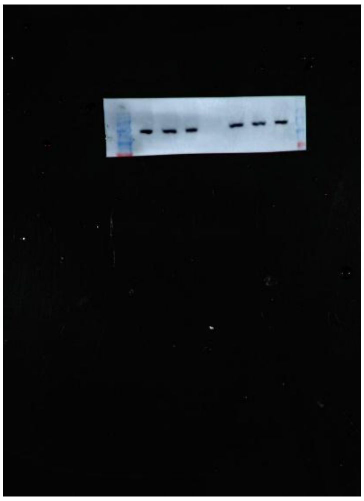

Fig.5B TRIB3

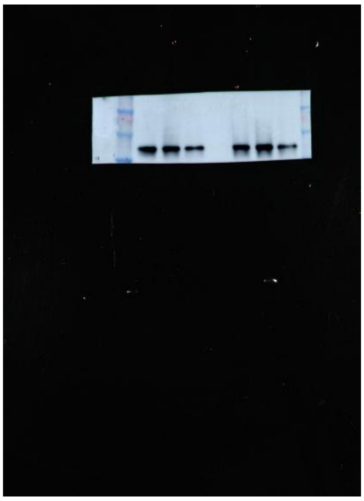

Fig.5B GAPDH

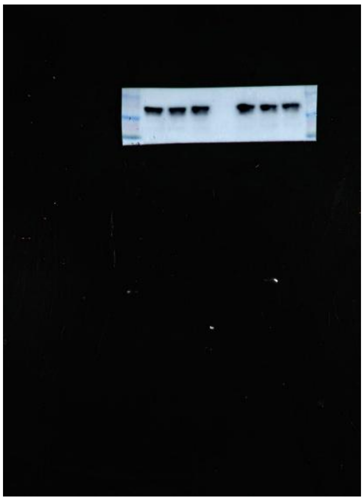

Fig.5C p-Src

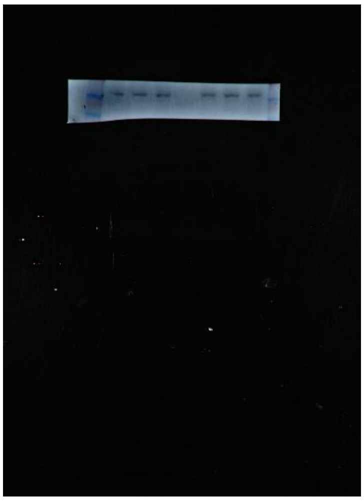

Fig.5C Src

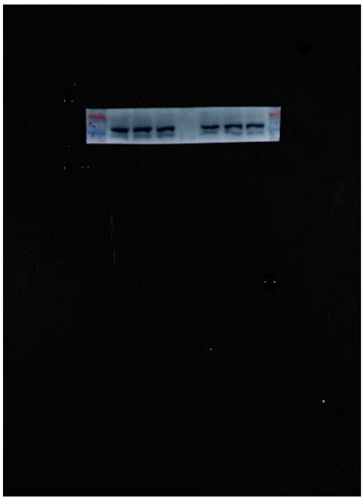

Fig.5C TRIB3

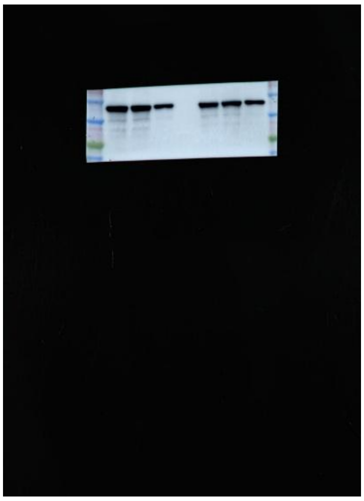

Fig.5C GAPDH

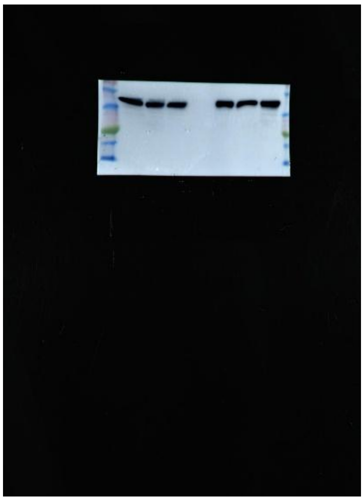

Fig.5D

HS746T

5-FU (μM)	Solvent control			Src inhibitor 1			Y15		
2	82.3	101.2	87.7	83.36	85.15	82.21	80.55	83.66	86.67
4	93.6	74.2	81.6	70.85	75.53	85.36	72.33	73.69	75.88
8	70.7	77.1	75.2	58.25	61.23	59.96	65.66	64.35	66.77
16	65.3	63.1	56.2	48.26	49.99	47.63	57.12	54.43	58.59
32	45.2	43.5	38.6	30.25	35.67	28.88	34.33	32.25	39.04
64	35.2	33.2	31.7	15.23	20.14	21.11	26.35	24.28	28.34
128	26.1	29.7	20.5	5.84	8.98	10.65	10.23	13.36	15.25
256	10.82	19.95	13.63	2.58	4.96	6.37	6.33	5.26	4.38
512	5.99	10.12	12.23	0.66	1.02	3.33	3.25	5.15	5.36

MKN-45 OE-PTPRE

5-FU (μM)	Solvent control			Src inhibitor 1			Y15		
2	92.25	85.56	81.33	72.65	72.36	74.62	75.58	82.33	85.61
4	72.36	75.36	70.17	55.69	49.96	53.61	61.66	63.59	65.32
8	60.58	53.36	56.69	33.69	35.25	32.33	37.12	39.64	36.94
16	42.25	44.59	40.18	25.16	27.18	22.38	28.28	30.13	25.92
32	31.15	26.69	29.01	13.26	10.24	11.96	16.68	18.25	16.66
64	19.19	26.63	16.67	9.16	8.17	12.22	12.36	14.56	18.29
128	16.36	15.25	15.98	8.78	5.25	3.95	11.15	10.36	12.36
256	7.13	16.25	12.21	3.36	2.25	1.16	5.15	2.63	5.66
512	6.86	5.29	7.95	0.23	1.22	0.68	3.26	4.16	5.25

Fig.5E

	Solvent control			Src inhibitor 1			Y15		
**HS746T**	23.88	21.06	25.64	15.06	17.77	13.03	18.02	19.19	17.06
**MKN-45 OE-PTPRE**	8.923	7.72	9.99	3.508	3.36	3.79	3.429	4.35	2.86

Fig.6A

HS746T

5-FU (μM)	si-Control			si-TRIB3			si-TRIB3+Fer-1		
2	92.5	94.6	86.2	86.3	88.2	84.1	90.6	93.5	88.5
4	82.5	84.7	80.3	74.2	76.6	80.3	83.5	79.6	81.3
8	73.6	74.1	76.6	60.5	62.8	61.1	75.5	72.9	72.3
16	62.1	64.4	59.2	41.7	46.2	45.3	57.3	61.8	60.7
32	43.3	43.6	40.7	32.6	33.1	29.5	41.2	40.6	37.2
64	33.1	31.3	30.2	23.3	22.8	24	30.6	28.2	29.1
128	25.8	27	24.3	9.1	13.2	14.7	20.2	18.9	17.6
256	16.2	17.1	15.8	9.3	8.2	7	13.3	10.6	12.5
512	13.1	8.8	11.2	2.5	3.2	2	10.1	9.2	7.6

MKN-45 OE-PTPRE

5-FU (μM)	si-Control			si-TRIB3			si-TRIB3+Fer-1		
2	85.2	83.5	79.2	70.6	68.2	65.5	83.4	82.1	78.6
4	69.3	73.2	67.7	50.1	47.3	51.6	65.1	68.2	66.9
8	57.3	51.3	54.5	28.2	29.1	31.3	49.2	50.3	51.5
16	40.1	42.6	38.3	23.2	24.2	19.9	37.6	35.5	36.1
32	30.2	27.5	28.8	13.7	12.2	12.5	28.2	26.5	27.3
64	20.3	23.5	19.1	10.8	10.6	11.2	15.1	19.2	17.7
128	15.8	18.1	16.2	9.9	6.3	5.8	10.3	13.8	15.2
256	12.6	13.3	11	5.3	4.6	4.2	7.5	9.3	8.6
512	10	7.2	7	3.3	2.5	1.9	5.2	7.3	5.1

Fig.6B

	si-Control			si-TRIB3			si-TRIB3+Fer-1		
**HS746T**	21.92	22.34	23.22	13.52	12.861	13.668	21.19	20.525	19.616

**MKN-45 OE-PTPRE**	8.496	8.931	8.132	2.693	3.01	2.27	7.686	7.832	7.53

Fig.6C

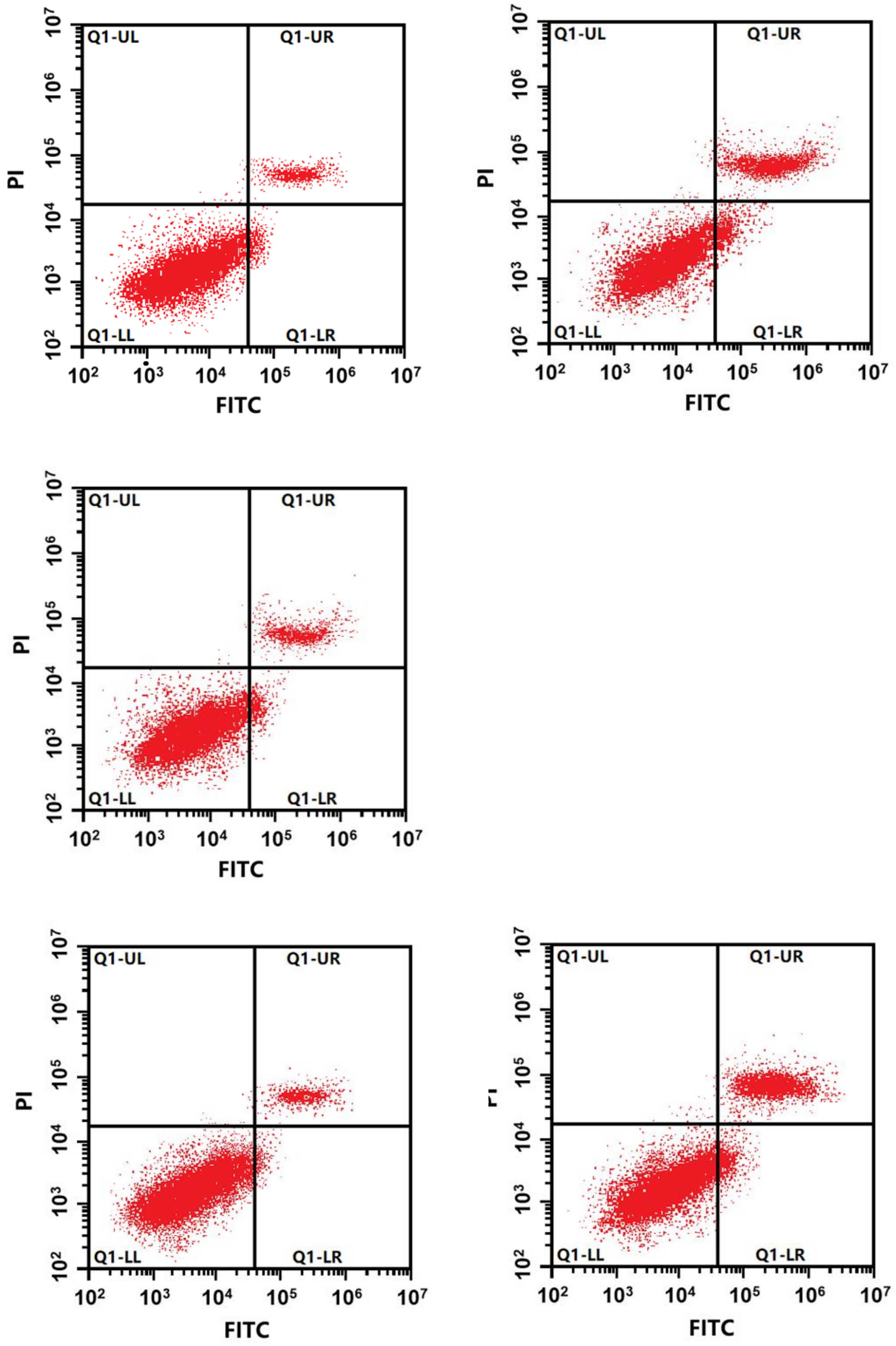

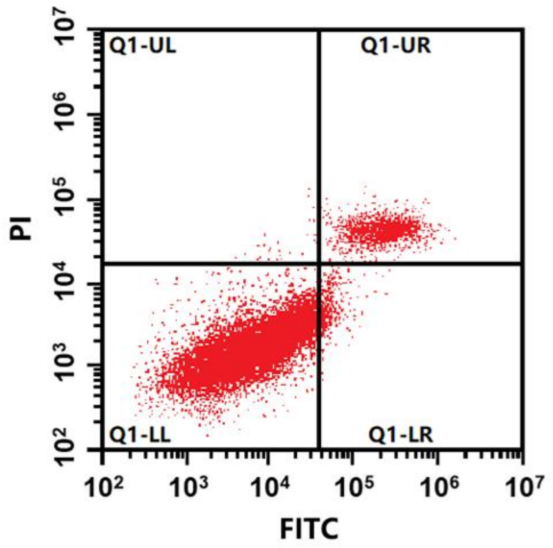

Fig.6D

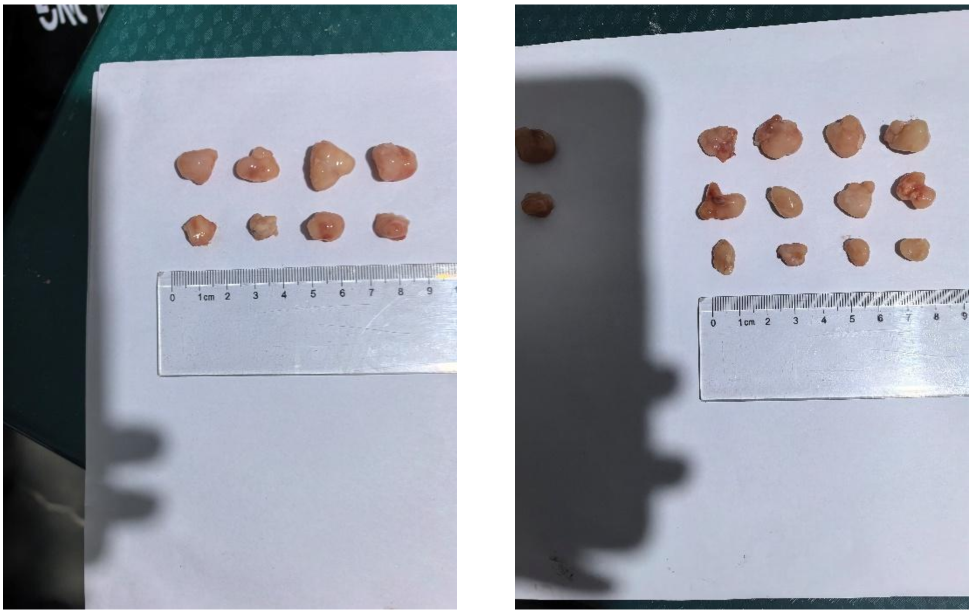

Fig.6E

Days	Control				5-FU			
1	116.3	118.9	120.1	123.1	117.2	118.6	121.3	122.5
3	150.6	153.2	165.1	170.2	130.7	138.6	137.1	135.8
5	194.8	196.2	209.3	205.7	150.2	163.2	165.7	182.7
8	233.6	240.1	263.5	284.3	164.3	180.2	178.6	201.7

**10**	298.2	305.3	333.2	346.2	185.2	201.8	198.5	221.9
**12**	363.2	369.1	421.7	430.6	207.5	219.6	218.7	249.6
**15**	479.6	483.7	525.6	538.9	226.5	233.1	238.7	268.9
**17**	555.2	567.2	613.5	673.2	257.4	260.7	266.5	295.3
**19**	682.3	703.1	759.6	833.7	288.6	292.5	297.6	325.1
**21**	776.5	801.2	846.2	925.8	308.6	322.2	329.6	349.6

**Days**	Control				5-FU				Erastin+5-FU			
**1**	118.6	121.3	122.2	125.7	118.7	120.4	122.7	125	119.2	119.8	123.1	124.3
**3**	158.1	160.3	164.6	169.7	150.6	154.3	152.6	165.1	128.6	130.3	131.5	134.6
**5**	196.7	199.2	206.3	202.3	170.2	185.1	199.3	203.6	140.2	150.6	155.1	161.7
**8**	239.6	246.3	258.6	277.2	210.6	218.8	216.6	236.2	165.3	170.6	180.2	186.3
**10**	302.3	320.1	327.1	358.2	249.3	255.2	278.8	321.7	191.2	203.2	212.1	223.2
**12**	383.6	380.5	398.7	423.1	314.3	321.2	335.5	378.5	206.7	228.1	237.4	261.2
**15**	483.2	486.8	496.5	525.36	382.6	398.3	413.3	432.9	213	234.6	241.3	275.1
**17**	572.1	582.3	593.8	715.2	455.6	486.8	505.3	589.2	243.1	249.1	256.7	281.6
**19**	695.6	713.7	732.5	860.2	560.3	562.5	596.8	698.3	268.2	273.5	281.3	305.1
**21**	803.8	825.2	843.6	981.5	669.2	680.6	721.3	799.8	298.6	303.6	310.7	336.2

Fig.6F

Control	5-FU
1.065	0.511
0.836	0.496
0.761	0.525
0.732	0.453

Control	5-FU	Erastin+5-FU
1.102	0.772	0.331
0.952	0.701	0.278
0.866	0.688	0.296
0.733	0.563	0.269
